# Androgen Insensitivity Syndrome: A rare genetic disorder

**DOI:** 10.1016/j.ijscr.2020.01.032

**Published:** 2020-02-06

**Authors:** Sushrut Fulare, Satish Deshmukh, Jyoti Gupta

**Affiliations:** NKP Salve Institute of Medical Sciences, Nagpur, India

**Keywords:** Androgen Insensitivity Syndrome, Genetic disorder, Karyotype mapping orchidectomy, Hormonal substitution therapy, Psychological counselling

## Abstract

**Background:**

Androgen Insensitivity Syndrome (AIS) is a rare X-linked recessive androgen receptor (AR) disorder with 46XY karyotype. Partial AIS affects 5–7 per 1,000,000 genetically male individuals whereas Complete AIS affects 2–5 per 100,000 genetically male individuals. CAIS CAIS is characterized by complete resistance to the action of androgens.

**Presentation of case:**

17-year patient presented with swelling in bilateral inguinal region. Patient also complained of primary amenorrhea with serum FSH and LH levels being raised, serum testosterone level much above normal range. MRI Pelvis revealed agenesis of vagina, uterine body, both ovaries and cervix. Bilateral testes were noted in bilateral superficial inguinal ring. Bilateral orchidectomy was done and the patient was advised estrogen substitution therapy.

**Discussion:**

CAIS is usually diagnosed at puberty, when the patient presents with primary amenorrhea. Karyotype has to be mapped in order to differentiate from other genetic disorders. Orchidectomy should be done to avoid risk of malignancy of undescended intra-abdominal testes (3.6 % at 25 years old, and 33 % at 50 years old, reported by various studies). Hormonal substitution therapy should be administered. Comprehensive psychiatric assessment and intervention go a long way in alleviating distress and enhancing quality of life.

**Conclusion:**

Androgen Insensitivity Syndrome requires expert and sympathetic handling. Close collaboration between surgeon, gynaecologist and psychologist is essential for proper management of complete androgen insensitivity syndrome.

## Highlights

• Androgen Insensitivity Syndrome is a rare X-linked recessive genetic disorder caused by mutation in Androgen Receptor (AR) gene.
• Individuals with Complete Androgen Insensitivity Syndrome have 46XY karyotype and present as female appearance and bilateral undescended testis.
• 17 year old patient, presented with swelling in bilateral inguinal region and primary amenorrhea.
• Karyotype was mapped as 46 XY, elevated serum testosterone level.
• Bilateral orchidectomy was performed and patient was advised hormonal substitution therapy and provided psychological counselling.

## 1 Introduction

Androgen insensitivity syndrome (AIS) is a rare X-linked recessive androgen receptor (AR) disorder [2]. The individuals with Androgen Insensitivity Syndrome have 46XY karyotype. AIS could be classified as complete, partial, or mild AIS according to the gradation of androgen insensitivity. Complete AIS (CAIS) is characterized by complete resistance to the actions of androgens. It is presented as female appearance and normal breast development, absence of uterus and ovaries, bilateral undescended testis, and elevated testosterone levels. Complete Androgen insensitivity syndrome is a rare disorder affecting 2–5 per 100,000 genetically male indivuals while partial AIS affects 5–7 per 1,000,000 genetically male individuals [1].

The following work has been reported in line with the SCARE criteria [3].

## 2 Case report

17 year old patient presented with swelling in bilateral inguinal region since 10 years. She also gave history of primary amenorrhea. Patient had poor secondary sexual characteristics, with breast developed at Tanners Stage II and genital and axillary hair development at Tanners Stage I. Patient did not have hoarseness of voice, receding hair line or white per vaginal discharge. Patient was normotensive with height of 163 cm, weight 55 kg, body mass index 20.75 kg/m^2^, waist circumference 78 cm, and hip circumference 86 cm.

The patient was brought up as a girl child.

Clinical examination revealed a female phenotype; the breasts were normally developed with scanty axillary and pubic hair. Per vaginal examination revealed a blind ending short vagina (2 cm). A solitary swelling of 2 × 2 cm was present in left inguinal region. Another swelling of 2 × 3 cm was present in right inguinal region. Both the swellings were soft, non tender, freely mobile, non reducible and Cough impulse test was negative.

Hematological Investigation revealed serum FSH and LH on the upper limit, elevated serum testosterone level, normal serum estradiol (E2) and serum prolactin levels, and slightly raised serum TSH level. Blood sugar level and lipid profile were within normal limit. In MRI Pelvis vagina, uterine body, both ovaries and cervix were not visualized, suggestive of agenesis. Bilateral testes were noted in bilateral superficial inguinal ring.

After counselling the patient and her relatives about the patient’s condition, the patient consented to identify herself as female and underwent bilateral orchidectomy with removal of the undescended testes also in order to avoid risk of malignancy. The testes were found inside the superficial inguinal ring. The specimen was sent for histopathological evaluation, which revealed two testes with atrophic seminiferous tubules containing only sertoli cells, associated to leydig cells hyperplasia.

The development and upbringing of the patient was that of a female and after the recent course of events the patient opted for psychiatric counselling. The patient was also advised hormonal substitution therapy with its course provided by gynaecologist.

## 3 Discussion

During embryological development the internal genitalia consist of two accessory ducts; the mesonephric duct (Wolffian duct) and the para-mesonephric duct (Mullerian duct). The mesonephric duct is the precursor to male genitalia and its development. Under the influence of SRY gene located on Y-chromosome, in the 7^th^ week of fetal development, the testes begin their differentiation [4]. Three key hormones responsible for male external and internal genital development are Anti-Mullerian Hormone, testosterone and dihydro-testosterone are secreted by the testes. The anti-mullerian hormone causes para-mesonephric duct to regress while testosterone influences the growth of male accessory structures i.e, penis, vas deferens, epididymis, seminal vesicle [5].

Androgen insensitivity syndrome is a X-linked recessive genetic disorder caused by mutation in the Androgen Receptor (AR) gene. Due to this mutation in AR gene, during fetal development, the cells fail to respond to androgen hormones thus preventing masculinization of male genetalia. It also prevents development of male secondary sexual characteristics during puberty [7].

Androgen Insensitivity Syndrome (AIS) is classified into complete androgen insensitivity syndrome (CAIS), partial androgen insensitivity syndrome (PAIS) and mild androgen insensitivity syndrome(MAIS) based on phenotype [2]. Other names given to various presentations of AIS are Reifenstein syndrome, Rose-water syndrome, Morris’ syndrome, Gilbert-Dreyfus syndrome, Goldberg-Maxwell syndrome, Lubs syndrome, Aiman’s syndrome [14].

Clinical features in complete androgen insensitivity syndrome are absent or rudimentary mullerian structures i.e, uterus, fallopian tube, cervix and a blind ended short vagina; two non-dysplastic, undescended testes; absence or scanty pubic hair and axillary hair; normal to underdeveloped breast. Family history would reveal a X-linked pattern inheritance with affected male individuals and manifesting heterozygous females (46, XX), although no significant family history does not preclude the diagnosis of CAIS [8,14].

Androgen Insensitivity Syndrome is most commonly diagnosed at puberty when the patient presents with primary amenorrhea. It can also be diagnosed prenatally with dissimilarity between the karyotype mapped from amniotic fluid and genetic sex verified on ultrasound. In rare cases the diagnosis is made in later period of life when the patient presents with infertility. Although no formal diagnostic criteria for identifying AIS has been established yet, karyotype mapping, elevated level of serum testosterone, normal or elevated level of serum FSH, LH and estradiol, radiological imaging of pelvis and molecular genetic testing showing mutation in AR gene helps in the diagnosis of AIS. Karyotype mapping holds maximal importance as it also helps to differentiate AIS from other genetic abnormalities like Klienfelters syndrome (47, XXY), Turners syndrome (45, XO), Mixed gonadal dysgenesis (45,XO; 46 XY), Tetragametic chimerism (46,XX; 46,XY) [6].

There is no definitive treatment to correct AR gene mutation as of yet, treatment is mainly based on symptomatic management [11]. Assignment of sex of rearing holds paramount importance. After evaluation, the patient along with parents or guardian should ne explained about the condition and an informed decision should be made. Majority of patients are raised as girl-child since birth and keep seeing themselves as woman. Furthermore, orchiectomy should be done to avoid risk of malignancy of undescended testes [10] (3.6 % at 25 years old, and 33 % at 50 years old, reported by various studies). Although rare, some females also choose to undergo vaginoplasty for creation of a functional vagina, similar results can be achieved by vaginal dilators. Patients are further benefitted by hormone replacement therapy. Also calcium and vitamin D supplementation with regular DEXA scanning for bone density is also advised. Those who choose to identify themselves as male can benefit from sex re-assignment surgery.

### 3.1 Psychological aspect of Androgen Insensitivity Syndrome

The reason for reporting this case is the rarity of complete androgen insensitivity syndrome and its multi-disciplinary management. Most of the patients with androgen insensitivity syndrome are brought up as a girl-child. For them the realization that they are genetically male and have only partial female characteristic and would not be able to bear children can be mentally devastating. As for few who are raised as male-child can benefit from sex re-assignment surgeries [9].

Our patient was raised as a girl child from birth. She was tall, had normal breast development for her age, scanty pubic and axillary hair. She also mentioned that she used female skin care and beauty products and was attracted towards male gender. The revelation of being genetically male was not very well accepted by her or her family at first and resulted in significant psychological trauma. It also resulted in minor depressive episodes. Our patient achieved satisfactory level of adjustment to assignment of feminine role after proper education and counselling. Although some doubt persisted in her mind about societal acceptance of her condition, also if she will be able to carry out her feminine duties well as she was made aware that she did not have child bearing capacity.

Androgen insensitivity syndrome can be extremely distressful to the concerned individual. Apart from orchidectomy, hormone replacement therapy, sex re-assignment surgery, comprehensive psychiatric assessment and counselling can go a long way in alleviating distress as well as improving the quality of life of affected individual [12,13].

## 4 Conclusion

To conclude, Androgen Insensitivity syndrome although rare, is very distressing to the individual and family members. It requires expert and sympathetic handling. Close collaboration between a surgeon, gynaecologist and psychiatrist is essential for proper management of complete androgen insensitivity syndrome. Due to the risk of degeneration of the gonad and malignancy of undescended testes, orhidectomy should be performed. The patient is also benefitted by hormonal replacement therapy as well as proper psychiatric counselling [8,10,11].

## Sources of funding

None.

## Ethical approval

Yes, NKP Salve Institute of Medical Sciences.

IEC/NKP SIMS-2/2018.

## Consent

Written informed consent was obtained from the patient for publication of this case report and accompanying images. A copy of the written consent is available for review by the Editor-in-Chief of this journal on request.

## Author contribution

Main operating surgeon: Dr. Satish Deshmukh.

Assitant operating surgeon: Dr Sushrut Fulare.

Second assisting surgeon: Dr. Abhiram Mundle, Dr. Jyoti Gupta.

Data collection: Dr Jyoti Gupta.

Writing: Dr. Jyoti Gupta, Dr Abhiram Mundle.

## Registration of research studies

Name of the registry:

Unique identifying number or registration ID:

Hyperlink to your specific registration (must be publicly accessible and will be checked):

## Guarantor

Dr. Satish Deshmukh.

## Provenance and peer review

Editorially reviewed, not externally peer-reviewed.

## Declaration of Competing Interest

None.
